# Cluster Case of Prolidase Deficiency: Varied Clinical Presentations and Management in a Sibling Trio

**DOI:** 10.7759/cureus.50661

**Published:** 2023-12-17

**Authors:** Saud Azhar, Palwasha Khan, Fahad Faizullah, Sohana Hakim, Madiha Aslam

**Affiliations:** 1 Internal Medicine, Hayatabad Medical Complex Peshawar, Peshawar, PAK; 2 Dermatology, Hayatabad Medical Complex Peshawar, Peshawar, PAK

**Keywords:** autoimmune disease, facial malformations, foot ulcer, pepd gene, collagen degradation, non-healing ulcers, prolidase deficiency

## Abstract

Prolidase deficiency (PD) is an exceptionally rare autosomal recessive disorder characterized by impaired collagen degradation, leading to the accumulation of proline-containing dipeptides. We report a cluster case of three siblings aged 17, 19, and 20 years, comprising of two sisters and one brother, who presented with non-healing ulcers on their shins and feet along with facial features of hypertelorism, depressed nasal bridge, reduced intellectual function, and high-arched palate. History and clinical features were consistent with PD. Due to the rarity of the disease and low socioeconomic background of the patients, specialized investigations or treatments were either unavailable or inaccessible. Furthermore, surgical intervention was ill-advised. Despite these challenges, patients were treated using improvised tailored therapy using three different modalities and showed remarkable progress. The evaluation and management took place at the dermatology unit of Hayatabad Medical Complex in Peshawar, Pakistan.

## Introduction

Prolidase deficiency (PD) is a rare autosomal recessive metabolic disorder caused by mutations in the peptidase D (PEPD) gene. It results in deficiency of the enzyme prolidase, leading to the accumulation of proline-containing dipeptides and a spectrum of clinical manifestations. Dermatological manifestations are most common, featuring non-healing ulcers, usually on lower extremities. Apart from this, characteristic facies, mental subnormality, and hematological abnormalities are present [1]. It is estimated to occur in approximately one to two per million live births. Due to its rarity, it is considered one of the rarest metabolic disorders. A total of 175 cases have been reported worldwide, and only two individual cases have been reported in Pakistan [2]. There are no clinical practice guidelines for this condition, and it is managed symptomatically [3].

This case report describes a unique presentation of PD in three siblings, which has not been reported before, and discusses the clinical evaluation, diagnostic process, and tailored management, including three different treatment modalities with promising outcomes in challenging circumstances.

## Case presentation

History

Three siblings, aged 17, 19, and 20, presented to our dermatology OPD with a history of non-healing painful ulcers on their lower limbs and feet. These ulcers had been persistent, recurring and poorly responded to previous treatments, including antibiotics and wound care.

Patient 1

The brother, aged 20, presented with symmetrical ulcers that had developed bilaterally on his lower limbs over the past 20 days. His dermatosis began at the age of 10-12 as a small papule/vesicle, which gradually progressed into painful ulcers. Ulcers were relapsing-remitting. They would heal with post-inflammatory hyperpigmentation and thickened skin. The patient complained of pruritis in the lesions while in the healing phase. Ulcers would aggravate in summer season and partially relieve with oral/topical medication.

Patient 2

The 19-year-old sister presented with multiple ulcerated plaques on both legs, involving the feet, with yellowish to reddish fluid discharge. The lesions had been present for several months, but the total history of her problem extended over seven years. It started as a vesicle on her right leg and progressed to ulcers. Ulcers would partially heal with topical and oral medication and aggravated with sweating and exposure to heat.

Patient 3

The 17-year-old girl had a single ulcer on her left ankle region from the past eight months. It had aggravated over the past two months following mild skin trauma. She also complained of claudication, palpitation, and mild photosensitivity.

Common causes of ulcers were ruled out, and there was no other past medical history. The patients did not have any history of cellulitis, limb ischemia, prolonged standing, cyanosis, insect bite, weight loss, blood dyscrasias, neuropathy, usage of topical steroids, diabetes, or any major trauma. All siblings showed mental subnormality as they hardly communicated and could not comprehend the doctor’s commands but had no history of any developmental delay or seizures. The siblings were born to consanguineous parents with the father having epilepsy. They had a fourth younger female sibling who had no ailment.

Examination

On physical examination, the siblings displayed multiple large painful ulcers of variable sizes and shapes on the distal one third of the shins, dorsa of feet, and ankles. The ulcers had irregular violaceous borders and sloping margins, and the floor had purulent discharge and granulation tissue. Chronic inflammation and scarring were evident on the affected skin. The skin surrounding the ulcer was erythematous, hyperpigmented, sclerotic, and bound-down. There was no loss of sensation, reflexes, or motor function in the limbs.

Patient 1 had bilateral involvement of the distal one-third of the shins and feet, with the largest ulcer measuring 10x10 cm on the dorsum surface of the left foot and extending to the lateral malleolus. He also had restricted movements of the feet and pruritis over the healing areas of the ulcer. Patient 2 had multiple ulcerated plaques on both legs and feet, with the largest measuring 15x12 cm on the right foot. Patient 3 had a single large, ulcerated plaque on the lateral side of the left ankle, above and around the lateral malleolus measuring 10×8 cm.

**Figure 1:**
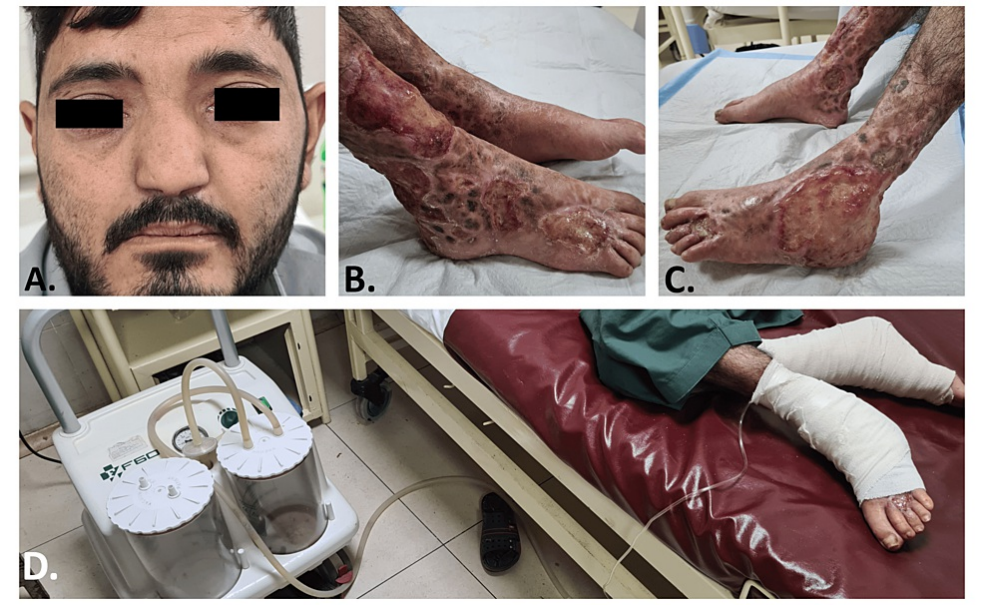
Patient 1 A. Facial dysmorphism showing prominent hypertelorism, proptosis, protruding and low-set ears, and depressed nasal bridge. B. Multiple large ulcers on the right dorsum of the foot extending behind the ankle and up to the shin. An irregularly shaped atrophic scar can be seen on the forehead. C. Single large ulcer seen on the left ankle and dorsum of the foot. D. Vacuum-assisted closure (VAC) dressing and vacuum suction machine can be seen applied to the feet.

**Figure 2:**
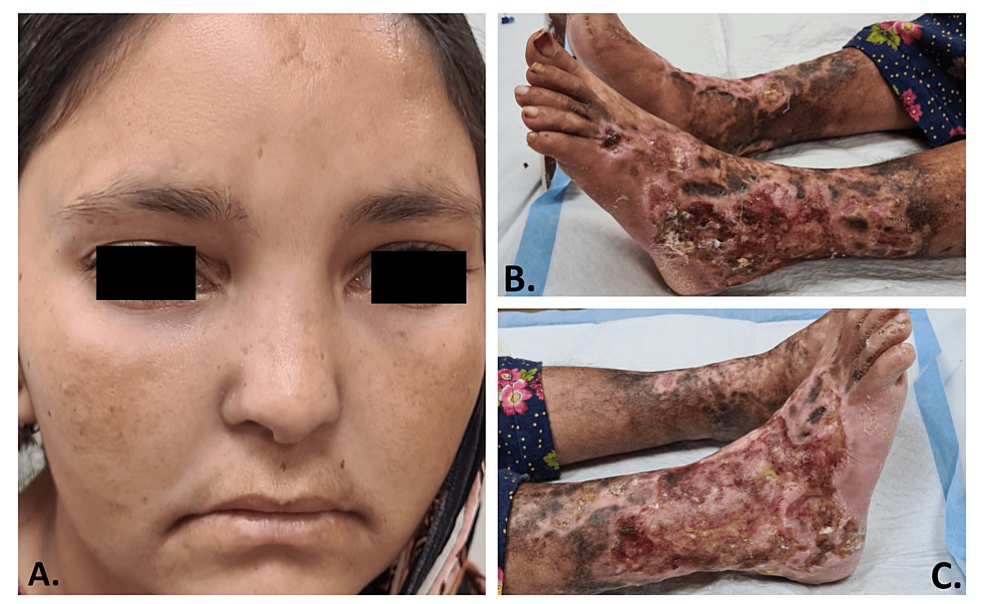
Patient 2 A. Facial dysmorphism showing a low set of ears, depressed nasal bridge, high forehead, hypertelorism, and mild pitted scarring. An atrophic scar can be seen on the forehead. B. Left foot showing a large irregular ulcer spread over the ankle with surrounding hyperpigmented skin. C. Large irregular ulcer on the dorsum of the right foot, extending laterally and superiorly to the shin. Interdigital scaling is also prominent.

**Figure 3:**
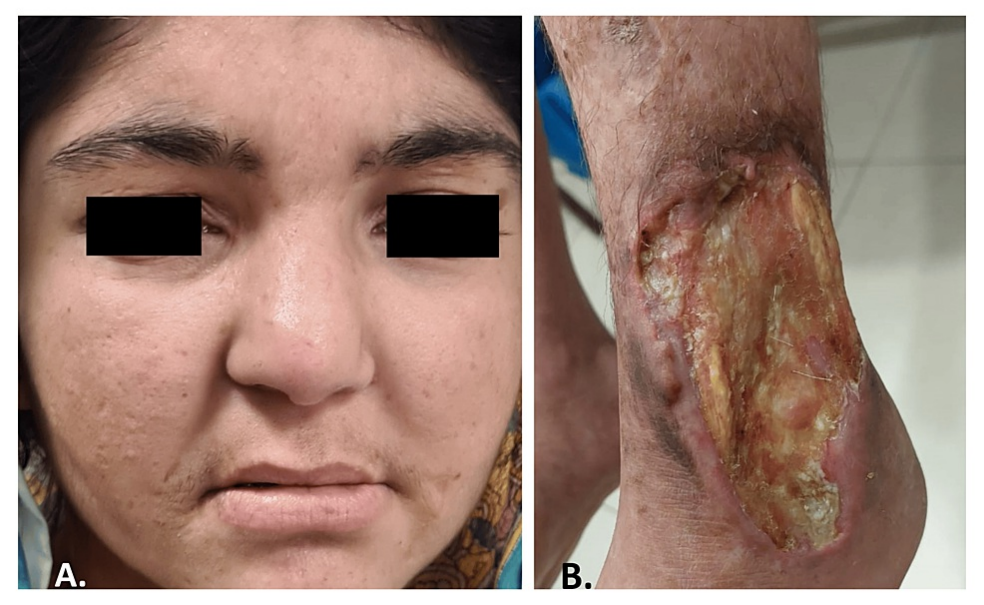
Patient 3 A. Facial dysmorphism showing a depressed nasal bridge, drooping eyelids, multiple pitted scarring around the mouth and on the cheeks, thinner vermilion of the upper lip, and prognathism. B. A single large ulcer on the left foot over and above the ankle can be seen with round borders and surrounding hyperpigmented skin.

The siblings also showed facial dysmorphism, including hypertelorism, high arching palates, malocclusions, low-set ears, micrognathia, and depressed nasal bridges. All patients showed mild facial scarring, with patient 3 showing prominent perioral pitted scars and some mild telangiectasias on acral parts of the body. Patients 1 and 2 also had a high forehead. Patient 1 showed mild proptosis, while patients 2 and 3 had mild bilateral ptosis. Mild premature graying of hair was noted. Figures 1-3 show the clinical features of patients 1, 2, and 3, respectively. All siblings had reduced cognitive abilities and speech-language impairment. Systemic examination revealed splenomegaly in patient 1 and mild thyroid swelling in patient 2. Blood pressure on average ranged 90-95/60-65 for all siblings with a regular feeble slow pulse rate.

Investigation and diagnosis

All siblings showed hypochromic microcytic anemia with anisocytosis. In addition, red cell morphology of patient 2 showed pencil cells and teardrop cells. Liver function tests were slightly deranged, as displayed in Table 1 along with other laboratory tests. The thyroid profile was normal in all siblings, but hypoalbuminemia was present. The urinalysis of patients 1 and 3 were normal, whereas patient 2 showed 1+ proteinuria. Local ultrasound showed mild subcutaneous tissue edema of the feet, while patients 1 and 3 also showed sluggish flow in the dorsalis pedis artery on color Doppler imaging. Pseudomonas was isolated from the wounds of all the patients. Chest X-rays were unremarkable.

**Table 1:** Laboratory reports Baseline investigations were done at the time of admission. Normal laboratory values taken from the Department of Pathology, Hayatabad Medical Complex, Peshawar, KPK, Pakistan. Hb: hemoglobin, WBC: white blood cell, Neut: neutrophils, ALT/GPT: alanine aminotransferase/glutamic pyruvic transaminase, ESR: erythrocyte sedimentation rate

Tests	Normal range	Patient 1	Patient 2	Patient 3
Hb, g/dL	11.5 – 17.5	11.4	9.07	11.1
Mean cell volume, fL	76-96	59.4	65.5	68.6
WBC, x10.e3/µL	4 – 11	13.5	9.35	9.55
Neut, %	40 – 75	80	75	62.8
Lymph, %	20 – 45	15	20	25.5
Platelets, x10.e3/µL	150 -450	248	281	174
Mean platelet volume, fL	7.2 - 11	7.69	10.5	8.67
Blood urea, mg/dL	18 -45	12.3	75	16
Creatinine, mg/dL	0.42-1.06	0.697	0.341	0.33
Total bilirubin, mg/dL	0.1-1.0	0.42	0.4	0.21
ALT/GPT, U/L	10-50	25.6	11	63.5
Alkaline phosphatase, U/L	40-129	111	112	131
ESR, mm/1^st^ hour	0 - 20	-	-	70
Albumin, g/dL	3.5 -5.5	-	2.9	-

Patient 1 also had mild bilateral conductive hearing loss, which was evaluated using pure-tone audiometry. Abdominal ultrasound of patients 2 and 3 were normal, while patient 1 revealed splenomegaly at 15 cm. Patient 2 showed a single enlarged right inguinal lymph node.

Differential diagnoses for non-healing ulcers and the associated features included primary immunodeficiencies, connective tissue disorders, and genetic syndromes. However, the combination of facial dysmorphism, non-healing pedal ulcers, splenomegaly, anemia, and developmental regression in these siblings confirmed the clinical diagnoses of PD. Molecular genetic testing and prolidase enzyme activity tests can be used to further evaluate the extent of disease, but they are not available in Pakistan unfortunately.

Management

Three specific treatment modalities were used for each case. This was done to suit each case and help evaluate the efficacy of each one. Glycine-proline preparations were not available in the region, and other treatment methods were unaffordable for the patients.

Patient 1

He received vacuum-assisted closure (VAC) dressing as his ulcers were the largest and difficult to heal. Dressing was placed for 48 hours for each session, with a total of three sessions per leg. The vacuum pressure was maintained at around 120 mmhg. Ulcers started healing and were covered with healthy granulation tissue after the six sessions (Figure 1D).

Patient 2

For wound care, hydrogen peroxide and normal saline washes were employed, and antibiotics were given according to the culture and sensitivity. After controlling the infection, the patient was started on steroid pulse therapy, and one pulse of IV dexamethasone 80 mg in 500 ml of 5% dextrose water for three consecutive days was given with regular monitoring of vitals and serum electrolytes. Along with the pulse therapy, supplements containing zinc 15 mg, iron 27 mg, manganese 5 mg, and ascorbic acid 90 mg were also given, one tablet a day for 30 days. The patient responded well to the treatment, and there was a marked decrease in the ulcers’ size and fresh granulation tissue started to appear. The patient was discharged with follow up advice after one week.

Patient 3

Patient 3 received conservative treatment as she had a smaller, solitary ulcer. Wound care included hydrogen peroxide and saline soaks, tetrachlorodecaoxide drops, and topical fusidic acid along with oral therapeutic multivitamins. Infection was controlled using 1.2 g IV co-amoxiclav, twice a day. The ulcer had reduced in size and was filled with granulation tissues by the time of discharge.

Follow-up and outcome

**Figure 4:**
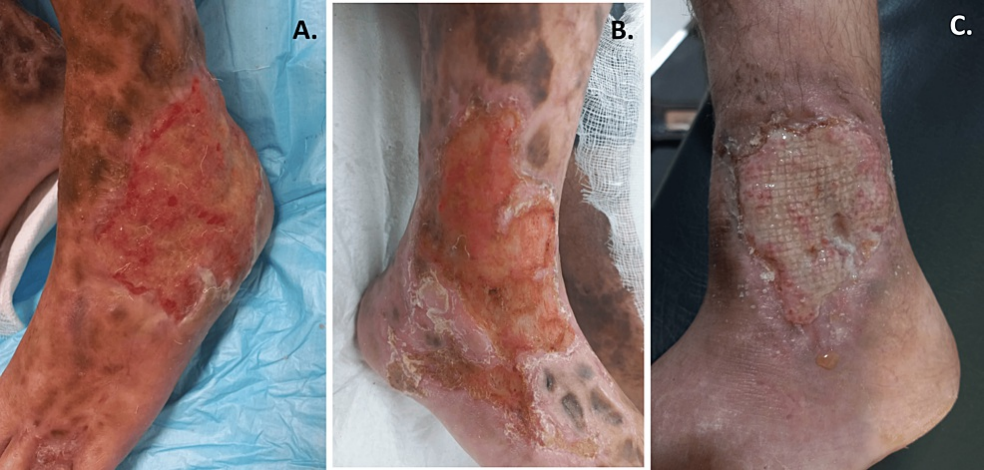
Follow-up images Images taken after two months of discharge. Image A: Patient 1 after vacuum-assisted closure (VAC) sessions. Image B: Patient 2 after pulse-steroid therapy. Image C: Patient 3 after the conservative treatment.

All patients came in for a follow-up first at two weeks and then after two months since discharge. They were examined thoroughly. Their ulcers showed progressing healing, with granulation tissues filled in the wounds. Ulcer sizes had reduced, with margins approximating. There was no signs of active infections, and no new ulcers were seen. The follow-up images are shown in Figure 4. Patient 1 was referred to plastic surgery for further management. Patient 2 was given another pulse of steroid on the second follow-up. Patient 3 was advised to continue taking multivitamins. All siblings were advised to continue regular follow-ups.

## Discussion

Clinically, PD is characterized by non-healing ulcers, distinctive facial features, intellectual impairment, hematological abnormalities, and splenomegaly, which are consistent with the presentation of our cases and two adult siblings from Lebanon with a long-standing history of the disease who were also children of consanguineous parents [3]. Recurrent ear infection was supported with the hearing loss in patient 1, but features, such as significant telangiectasias, thrombosis in the cutaneous microcirculation, or past history of respiratory infections, were not present, which were additional findings in a twins case of PD [4]. 

PD has been reported in twins and up to three newborn siblings in one case report before [4,5]. No case of three adolescent siblings has been reported yet with distinct pathognomonic features. Dermatological manifestations appear around six to 12 years of age, while neurological, pulmonary, endocrine, renal, and immunological manifestations, among others, appear before three years of age [6]. It should be noted that not all manifestations are present in all cases as evident by a case of a four-year-old Hispanic boy with pink, eczematous papules and plaques on antecubital fossa, popliteal fossa, and dorsal fossa along with dysmorphic facies [6].

Laboratory findings may include massive imidodipeptiduria, thrombocytopenia, elevated liver enzymes, hypocomplementemia, elevated immunoglobulin E levels, hypochromic microcytic anemia, and elevated erythrocyte sedimentation rate (ESR) values. Diagnosis can be confirmed through baseline investigations and clinical features [7]. Molecular and serological testing can be performed to measure the extent and variation of the disease, but they might not be available in certain socioeconomic settings. In such cases, clinicians should make the most out of the clinical examination, history, and available investigations to rule out any other cause.

Management remains challenging, with varying treatment outcomes reported but no established practice guidelines. Topical proline and glycine ointments, immunomodulatory therapies, topical tacrolimus and steroids, hyperbaric oxygen therapy, and anticoagulant treatments have been attempted with delayed partial healing of wounds and halting progression of ulcers, but there is no definitive cure as ulcers recur after some time, ranging from a few months to a year [3-8].

Our cases showed great outcomes with the different treatment strategies used for each patient, namely, VAC dressing, high-dose pulse steroid therapy, and conservative treatment along with infection control. These proved to be significant as they were readily available and affordable. However, the long-term outcome and recurrence of the ulcers remain to be seen. High-dose pulse steroid therapy has been previously shown to successfully treat ulcers in PD and ulcers recur less frequently [9]. Anticoagulant therapy is also suggested for improved wound healing and preventing thrombosis in its microcirculation, but in our cases, the Doppler study did not indicate any occlusions, and thus it was not required [10]. Multivitamins were given to patient 3 to prevent any nutritional deficiency that might delay wound healing. Conservative treatment, including antibiotics and wound care, heal ulcers and control infections but do not alter the course of the disease [6]. VAC and pulse steroid therapy were used on a three-year-old child with successful results, where surgical intervention was also necessitated due to a complication [11]. Furthermore, VAC has been proven as a useful adjunct in the management of benign chronic leg ulcers other than PD [12].

PD is an extremely rare disorder with only two previous cases from Pakistan [1,2], emphasizing the need for increased awareness and further research to improve diagnosis and treatment options for affected individuals.

## Conclusions

Our case documented the occurrence of PD in three siblings in their late teens. Their clinical features and laboratory findings were typical of the disease, namely, recurrent chronic ulcers in the lower limbs, dysmorphic facies, splenomegaly, anemia, and mild thrombocytopenia. Upon excluding all other causes of chronic ulcers, the diagnosis was established. Timely diagnosis is important to prevent recurrence or complication of ulcers and unnecessary investigations.

This case highlights a potential gap in identification of such rare conditions. Our report aims to assist future diagnoses and suggests that this disease may be more prevalent than assumed. Negative-pressure wound therapy and high-dose pulse steroid was beneficial, considering the resource-limited setting. However, long-term follow-up is required to evaluate the benefits.
